# Influence of weather conditions and projected climate change scenarios on the suitability of *Vitis vinifera* cv. Carignan in Rioja DOCa, Spain

**DOI:** 10.1007/s00484-022-02258-6

**Published:** 2022-03-11

**Authors:** M.C. Ramos, F. Martínez de Toda

**Affiliations:** 1 Department of Environment and Soil Sciences, University of Lleida-Agrotecnio CERCA Center, Av Rovira Roure 191, 25198 Lleida, Spain; 2 ICVV (Universidad de La Rioja, CSIC, Gobierno de La Rioja), c/ Madre de Dios, 51, 26006 Logroño, Spain

**Keywords:** Acidity, Anthocyanins, Climatic change, Phenology, Water availability

## Abstract

Grape development and its quality are highly dependent on soil and weather conditions. Under the progressive warming, which can affect the suitability of typical varieties grown in a given area, the knowledge of the vine response to changes in climate is essential to stablish strategies to maintain the viticulture sector. This research presents an analysis of phenology and grape composition of the Carignan cultivar, during a 13-year period, at two locations in Rioja DOCa. Based on the results obtained and the projected changes in climate under climate change scenarios (RC4.5 and RCP8.5), the response of this cultivar was evaluated. Differences in the phenological dates of up to 18, 29 and 40 days, for flowering, veraison and harvest, respectively, were observed between the warmest and the coolest years. An advance of up to 5, 8 and 11 days, respectively, for the mentioned stages, is projected under the RCP4.5 scenario by 2050, which could be near 1.5*times higher under the RCP8.5 scenario. These advances will be mainly driven by the temperatures recorded in the previous period. Grape acidity was mainly driven by water availability, in particular during ripening, which imply a slight projected reduction due to precipitation changes but not significant effect due to increasing temperatures. The phenolic composition could be positively affected by increasing temperatures and increasing water deficits, since this variety does not always reach a complete maturity at present. Thus, under the projected warming scenarios, the suitability of Carignan in Rioja DOCa was confirmed.

**Supplementary Information:**

The online version contains supplementary material available at 10.1007/s00484-022-02258-6.

## Introduction

Climate warming and the lack of water in some areas will challenge growing vines, in particular those varieties with earlier phenology and those less resistant to droughts. One of the main observed changes in vine development during the last decades has been the advance in phenology (Jones and Davis 2000; Bock et al. 2011; Tomasi et al. 2011; Webb et al. 2012; Ruml et al. 2016; De Cortázar-Atauri et al. 2017; Fraga and Santos 2017), which could be different for each variety and location. In addition, grape quality is also linked to the climatic conditions (Teixeira et al. 2013; Barnuud et al. 2014; van Leeuwen and Darriet 2016; Pons et al. 2017; Wenter et al. 2018), and both the changes in the weather conditions and the advance in the phenological stages can have negative effects on grape quality. The expected increase in temperatures may have significant impacts on sugar content (Greer and Weston 2010) and alcohol levels (Duchêne and Schneider 2005) but can also lead to a decoupling between sugar contents and phenolic compounds (Martínez de Toda and Balda 2015; Sadras and Moran 2012). On the other hand, vine water status can have significant impacts on berry weight (Ojeda et al. 2001) and shoot growth (Pellegrino et al. 2005), but also on other grape components such as acidity (Barnuud et al. 2014; Sweetman et al. 2014), anthocyanins and phenolic compounds (Greer and Weedon 2014; Pérez-Álvarez et al. 2021; Calderan et al. 2021).

Given the projected climate change scenarios, it is necessary to explore strategies to maintain the sustainability of the wine sector in a given area. In this respect, several authors have pointed out be the introduction of varieties that can be more resilient to the effects of climate change (Wolkovich et al. 2017; Suter et al. 2021). The vines need a certain period of time to be well stablished and give good quality grapes, and the velocity at which all these changes are happening suggests urgent actions to mitigate the potential effects for the wine producer sector. A first step could be exploring the suitability of already adapted varieties, cultivated at present as minority varieties, that could offer some advantages compared to the main varieties cultivated in the area, and analysing their potentiality under future warmer conditions.

The Rioja designation of origin is a winegrowing region, located in the north of Spain, covers about 65,700 ha of vineyards, that produce about 260 ML of wine annually under the Qualified Denomination of Origin ‘Rioja’ (DOCa Rioja), and its wines are worldwide recognised (more than 37% of the production goes to the international market). The area has a continental climate, although three areas are distinguished due to the Atlantic and Mediterranean influences: Rioja Alta (Atlantic influence), Rioja Oriental (Mediterranean influence) and Rioja Alavesa (intermediate conditions). The vines are cultivated at elevations that range between 300 m a.s.l. (terraces of the Ebro River) and about 700 m a.s.l. The average temperature in the growing season in the region varies between 16 and 18.8 °C, with higher temperatures recorded in Rioja Oriental than in Rioja Alta, particularly in the areas located at the lowest elevation. The Winkler index values ranges between 1275 GDD (in Rioja Alta) and more that 1800 GDD (in Rioja Oriental) and precipitation in the growing season is quite scarce (between 146 and 250 mm, on average depending on the zones) (Ramos and Martínez de Toda 2019). The main soil types in Rioja are Calcixerolic Xerochrept, Petrocalcic Calcixerolic, Calcic Haploxeralft, Typic Xerorthent, Litic Xerorthent and Litic Palexeralf (Gómez-Miguel 2006).

In the Rioja Designation of Origin (Rioja DOCa), 90% of the vines are planted with red varieties (mainly Tempranillo, Garnacha, Carignan (Mazuelo), Graciano and Maturana Tinta) and 10% with white varieties (Macabeo (locally named Viura), Malvasía de Rioja, Garnacha Blanca, Tempranillo Blanco, Maturana Blanca, Turruntés, Verdejo, Chardonnay and Sauvignon Blanc). Tempranillo is the main variety, covering about 80% of the total vine surface, and the most characteristic variety from the region. This grape variety, originally from Spain, is the red variety most cultivated in Spain and occupies the third position among the red varieties in the world for wine production, with 231,000 ha, only behind Cabernet Sauvignon and Merlot (OIV, 2017). Tempranillo has short growth cycle with an early ripening. It is sensitive to pests and disease and to extreme drought and high temperatures and it can produce wines with a good balance of alcohol content, colour, and an honest, smooth, fruity mouthfeel that turns velvety as it ages and that can withstand long ageing periods (Balda and Martínez de Toda 2017).

Under the projected changes in climate, the growing cycle of the vines can suffer an advance of the phenology and a shortening of the growing cycle, and Tempranillo having early phenology and a short cycle could be negatively affected (Ramos and Jones 2018; Ramos and Martínez de Toda 2020a; Ramos et al. 2021). For that reason, it is important to explore in more detail the behaviour or other varieties that may be suitable for the area under similar climatic conditions, to maintain the sustainability of the area. Among the other red cultivated varieties, we will focus on Carignan. It is another variety with Spanish origin, from the Aragon region, but cultivated in different viticultural regions around the world (France, Italy, Chile, USA…). It is known as Cariñena in Spain, Carignane in the USA, Carigano in Italy and locally named Mazuelo in Rioja. It occupies a world surface of around 73,000 ha (OIV 2017).

There is evidence that this grape variety has been grown in Rioja for several centuries, but today it barely covers 2% of the wine region’s vineyards. It is more productive than other red varieties, albeit particularly sensitive to powdery mildew and needs more heat summation to mature. Although short on flavours, it produces wines with abundant tannins, high acidity and stable colour, which makes it a good complement to Tempranillo for wines to be aged for long periods (Martínez de Toda 2018).

These facts could offer optimal characteristics to be grown under warmer conditions in contrast to what may happen to Tempranillo, which is a variety that produces wines of low acidity and in addition it may suffer a reduction in acidity and in total anthocyanin concentrations under increasing temperatures (Ramos and Martínez de Toda 2020a). Under this hypothesis, this research aims to contribute to the knowledge of the response (phenology and grape composition) of Carignan under different weather conditions at present and under projected warmer scenarios in Rioja DOCa. The vine response was analysed during a 13-year period, in which different weather conditions were recorded, at two locations with differences in the soil properties. Based on the results obtained during the period analysed and the projected changes in temperature and precipitation by 2050 and 2070 under climate change scenarios (RC4.5 and RCP8.5), some projections were done and compared to those already projected for the main cultivar (Tempranillo) in the DOCa.

## Material and methods

### Study area and vine information

**Fig. 1:**
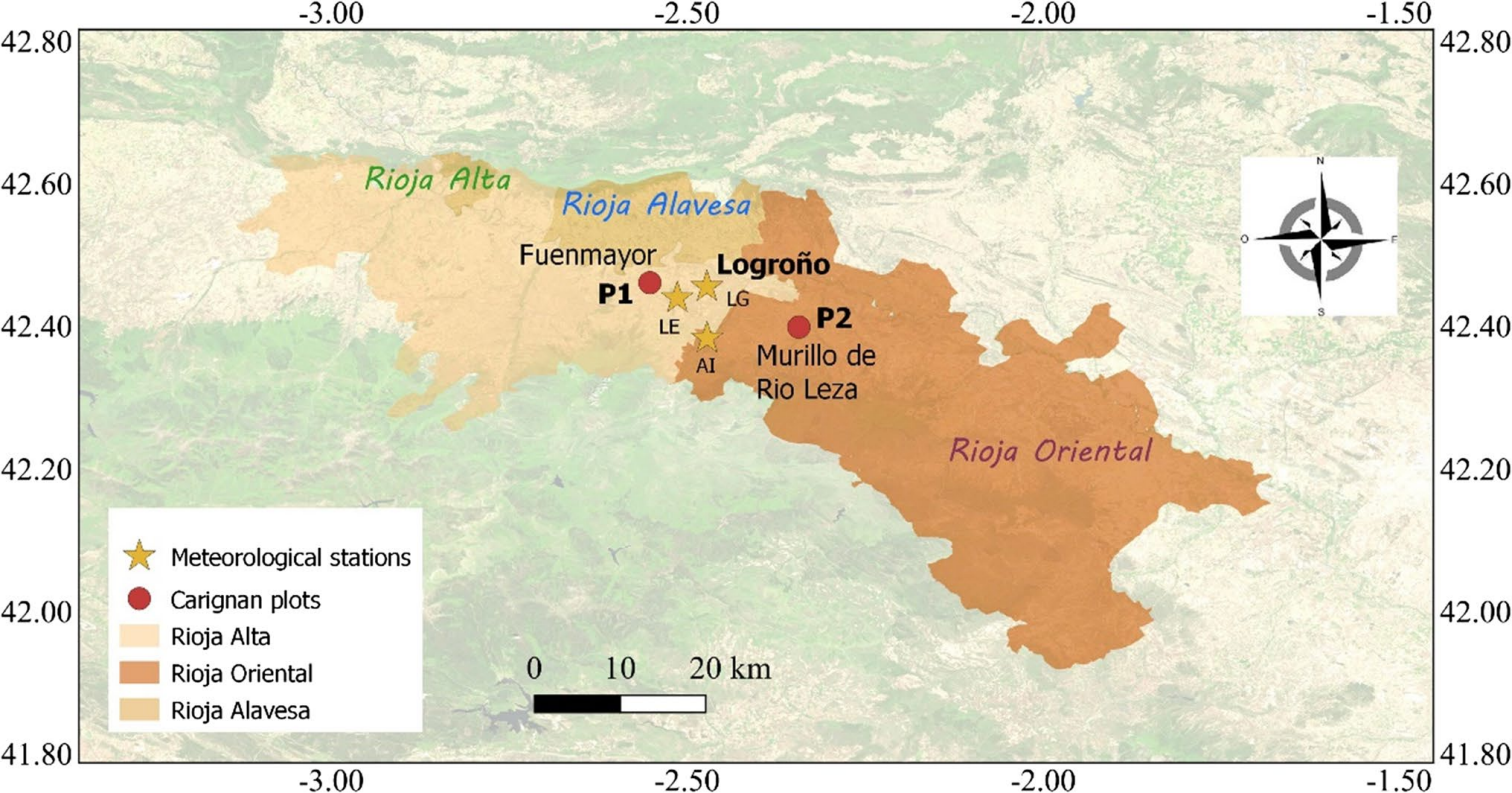
Location of the study area

The research was conducted in the central part of Rioja DOCa (Spain) in vineyards planted with *Vitis vinifera* cv. Carignan (Fig. 1). The information referred to two plots, which were located at 428 and 460 m a.s.l., and covered the period 2008–2020. The vines were planted in 1980 and 1986, respectively, in P1 and P2, on rootstock 110 Richter, and trained in trellis with a double cordon and a pruning of six spurs per vine, with two buds each. The planting pattern followed the regulations stablished by the Consejo Regulador of Rioja (3250 vines/ha with an average pattern of 2.8 m*1.1 m) and vines were not irrigated.

The dates at which 50% of the plants in the survey plots reached the phenological stages of flowers separated and veraison, corresponding to stages H and M according to Baillod and Baggiolini (1993), and the harvest dates (usually based on sugar contents), were considered. Grape sampling was carried out collecting berries in 40 randomly selected plants, at a rate of one cluster per plant and five berries per cluster from different parts (up, central and lower parts of the cluster). The grape composition [berry weight (BW), total soluble solids, expressed as probable alcoholic degree (PVAD), titratable acidity (AcT), malic acid (AcM), anthocyanins (AntT), polyphenols (TPI) and colour intensity (CI)] were evaluated weekly between veraison and maturity (between five and seven samples) in each year and location. All grape parameters were analysed in the Haro Oenological Station following the methods recommended by the OIV (OIV 2004). All vine information was supplied by the Consejo Regulador of Rioja DOCa.

### Climatic information: present and projections

The weather conditions during the period of analysis (2008–2020) were evaluated using daily data (maximum and minimum temperatures (Tmax and Tmin), precipitation (P) and potential evapotranspiration (ETo) estimated according to Penman Monteith) recorded in weather stations near the analysed vineyards (in Logroño: LE at 408 m a.s.l and LG at 456 m a.s.l; and in Albelda de Iregua:AI at 487 m a.s.l) (Fig. 1). These weather stations belong to La Rioja Government. The variability in temperature and precipitation with elevation was taken into account to better fit the conditions recorded at each analysed plot. The daily data were averaged for the growing season (GS: bud break-harvest) and for periods between phenological stages (BB-FS: bud break-flowers separated; FS-V: flowers separated-veraison; V-H: veraison-harvest). These variables were used to analyse the response of the vine under different weather conditions. During the period analysed, there was high variability in the weather conditions recorded during the growing season referred to both temperature and precipitation. This variability can be observed in Figure SM1, in which it is presented the information referred to the growing cycle (April 15th–October 15th) recorded in one meteorological station located in between the two analysed plots. The average maximum and minimum temperature recorded during the growing cycle was, on average, 24.7 ± 0.9 and 11.8 ± 0.8 °C, respectively, but with differences between years higher that 3 °C, for both Tmax and Tmin. The average precipitation during the growing season was 217 ± 55 mm, ranging between 131 and 308 mm (Figure SM1).

In addition, hourly temperatures, for the same period, were considered in order to evaluate the thermal requirements (chill and heat units during the dormant period and until reaching bud break). The chilling and warming periods were identified by analysing the daily chill and heat accumulations from the dormant period following the methodology described in Ramos (2017). Daily chill accumulation (in Chill Portions) was calculated according to the Dynamic Model (Fishman et al. 1987) using hourly temperature data, and heat accumulation (in Growing Degree Hours) was calculated according to Anderson et al. (1986), using a base temperature of 4 °C and an optimum temperature of 26 °C (Parker et al. 2011), from October 1st to April 30th. As the dates corresponding to bud break were not available for all years, a critical amount of chilling units (100 CHU) was considered, similar to that observed in previous research (Ramos 2017 and Andreoli et al. 2019). The heat units were accumulated when that threshold was reached in each year and the optimal base temperature (Tb) for each period was estimated following the methodology applied by Ramos (2017). A top threshold limit in the temperature was established (Tmax = 26 °C, the same that was already mentioned in the delimitation of chill and heat units). The agreement between the observed and predicted dates was analysed using the root mean square error (RMSE) (Eq. 1), and the Willmott index of agreement (d) (Willmott et al. 2012) (Eq. 2).

1 $$RMSE=\sqrt{\frac{\sum_{1}^{n}\left(DOYs-DOYo\right){ }^{2}}{n}}$$

2 $$d=1-\frac{{\sum_{1}^{n}(DOYs-DOYo)}^{2}}{{\sum_{1}^{n}\left[\left(DOYs-\overline{DOY }o\right)+(DOYo-\overline{DOY }o)\right]}^{2}}$$

where DOYs and DOYo are the simulated and observed dates at which the corresponding phenological event occurs, respectively.

The average heat accumulation value at which each phenological stage was reached for each variety was considered to determine the changes in timing under different climate change scenarios.

The projected climatic conditions under different emission scenarios (RCP4.5 and RCP8.5) were simulated using the MarkSim weather generator for 2050 and 2070. The average daily maximum and minimum temperature, and precipitation were projected using an ensemble of models (BCC-CSM1-1; BCC-CSM1-1-M; CSIRO-Mk3-6–0; FIO-ESM; GFDL-CM3, GFDL-ESM2G; GFDL-ESM2M; GISS-E2-H; GISS-E2-R; HadGEM2-ES; IPSL-CM5A-LR; IPSL-CM5A-MR; MIROC-ESM; MIROC-ESM-CHEM; MIROC5; MRI-CGCM3; NorESM1-M) (http://gisweb.ciat.cgiar.org/MarkSimGCM/ docs/ doc.html). The average of 20 simulations was considered. Taking into account the projected temperatures and precipitation as well as the thermal requirements to reach each phenological stage, the potential changes in the phenological timing was simulated. In addition, the potential changes in grape composition were also evaluated.

### Soil characteristics and its impacts on water availability

Soil characteristics of the study plots (soil particle distribution, gravels, organic matter content and soil depth) were considered to evaluate the available soil water in each year (ASW) in different periods along the growing cycle. The soil water storage capacity (SWC) and the ASW along the cycle were estimated using the VSIM model (Division of Science & Environmental Policy, California State University Monterey Bay Seaside, California), based on the soil water storage capacity at field capacity and the wilting point, estimated according to Saxton et al. (1986). The model was calibrated and validated in a previous work (Ramos et al. 2020). The ASW divided by the total soil water storage capacity (SWC) was used to evaluate the soil water deficit and stress level that suffered the vines (Pellegrino 2003). It was considered that 20–32% of SWC represented weak water deficit; 8–20% of SWC represented weak to moderate water deficit; 8–2% of ASWC represented moderate to severe water deficit, and values < 2% of SWC indicated severe water deficit.

### Grape composition variability related to temperature and water availability

The influence of temperature and available water on grape composition was evaluated in a multivariate analysis (factor analysis). The variables related to acidity (titratable acidity and malic acid) and to anthocyanins were included together with the average maximum and minimum temperatures in different periods (FS-V and V-H) and the average ASW in each period (simulated for each year as explained in the “Climatic information: present and projections” section.). The information referring each year for both plots was included in the analysis. The factors were extracted using principal component analysis (PCA) and the varimax rotation technique was used to maximise the variance shared among items and clarify the relationship among factors. An additional forward stepwise multiple regression analysis was done taking into account the variables that showed higher weight in each factor, in order to know the ones that could have higher influence and could produce higher effects under future changes in climate.

## Results

### Soil characteristics and soil water storage capacity

The soils of the analysed plots have clay loam textures, with 30.7 and 36% of sand, a clay content that ranges between 26.4 and 19.6% and a percentage of coarse elements (gravels) that ranged between 17.6 and 10.4%, respectively, in plots P1 and P2. The organic matter contents were relatively low (between 1.5 and 1.8%). Based on this information, the soil water content at field capacity ranged between 28 and 31% while that corresponding to the wilting point was 12 and 15%, respectively. Taking into account this information, the average ASW was estimated. It was observed that the ASW varied along the growing cycle, reaching very low values (< 5% of the SWC) at the end of the growing cycle (corresponding to the period veraison to maturity (Figure SM2).

### Phenology variability during the period analysed and its relationship with the weather conditions

Vine phenology referring the stages H (flowers separated), M (veraison) and harvest presented high variability between years. The dates at which the stage H was reached varied between May 17th and June 5th (average: May 25th ± 5 days); veraison dates ranged between August 2nd and 31st (average: Aug. 19th ± 6 days) and harvest dates varied between September 1st and October 12th (average: Sep. 30th ± 9 days). There were no significant differences between both plots within a given year. The major differences were observed in the harvest date under the most extreme conditions, as it was the case of the years 2008 or 2017, with earlier harvest (about 10 days earlier in plot P2 than in plot P1). An advance trend was observed for veraison and harvest (0.93 and 0.83 days per year, respectively).

The observed trends in the phenological dates were mainly driven by the differences in temperatures recorded in the previous period. As it was indicated before, there was high variability in the dates at which the stage H was reached, and although there was a slightly advance with increasing Tmin values in the previous period, the relationship was not significant. The date at which veraison was reached was affected by temperature recorded in the period between bloom and veraison (with an advance of about 2.7 days per an increase of 1 °C in the mean temperature recorded in that period), but there was also an accumulated effect of the temperatures recorded before bloom (as denotes the correlation with the temperatures recorded in that period). Similarly, maturity was also affected by temperatures recorded in the previous period (between veraison and maturity) with an advance of about 3.65 days per an increase of 1 °C in the average temperature recorded in that period, but it was also affected by the temperatures recorded in the previous periods (Table SM1). Despite the fact that later phenological dates were observed in the wettest years, which was in agreement with the sign found in the correlations with ASW in different periods (Table SM1), its effect on the phenological timing was smaller than that of the temperature. The wettest years recorded also lower temperatures and it seemed that the temperature was the main driver of the variability in the phenological timing.

In addition to the previous analysis and in order to deepen the effect of temperature on phenology of this variety, the thermal requirements to reach each phenological stage were analysed. The accumulated 100 chill units, considered the threshold to start to accumulate heat units, was reached on average between the 15th and 25th March, which was in agreement with results in other zones in the Rioja DOCa and in other close viticultural areas in Spain (Ramos and Jones 2018; Ramos and Martínez de Toda 2020a). Based on this information, heat (GDD) was accumulated from March 15th and the base temperature for each stage was then estimated. The base temperatures were 7.4, 6.3 and 5.2 °C, for flowers separated, veraison and maturity, respectively, and taking into account those temperatures, the average GDD needed to reach the corresponding stages was 1350, 1690 and 2480 GDD, respectively. The goodness of the fit was analysed using the root mean square error (RMSE) and d-Willmott index values (RMSE: 6.82, 5.16 and 8.71; d-Willmott index: 0.57, 0.87, 0.68, for flowers separated, veraison and maturity, respectively), which according to the criteria given by Moriasi et al. (2007) indicated moderate to good agreement between simulated and observed dates. The average GDD values were used to project the changes in the phenological dates under warmer scenarios.

### Grape composition variability during the period analysed

During the period of analysis, there were differences in the grape composition from year to year and also some differences between both plots, although the values followed similar trend in both plots. Grape composition at harvest in each plot and in each analysed year is shown in Table 1. The sugar content at harvest was slightly greater in plot P2 than in plot P1 while the average berry weight was higher in plot P1 than in plot P2. The titratable acidity presented high variability among years, with slightly greater values in P1 than in P2. Higher differences existed in the malic acid concentration between both plots. The anthocyanin concentrations, TPI and CI at maturity also showed high variability among years, with higher values in the dry and warm years (such as 2009, 2011, 2012 or 2017) than in years with other climatic characteristics, but with higher values in plot P2 than in plot P1. The differences in acidity, sugar content and anthocyanin concentrations were coherent with the different berry weight found between the plots, which could be related to the differences in the available water.

**Table 1:** Grape composition at harvest in the two analysed plots ( P1 and P2) planted with Carignan in the period 2008–2020 (*BW*, berry weight of 100 berries; *PVAD*, probable volumetric alcoholic degree; *AcT*, titratable acidity; *AcM*, malic acid; *AnT*, total anthocyanins; *TPI*, total polyphenol index; *CI*, colour intensity)

	BW-100b (g)		PVAD (º)		AcT (g/L)		AcM (g/L)		AntT (mg/L)		TPI		CI	
	P1	P2	P1	P2	P1	P2	P1	P2	P1	P2	P1	P2	P1	P2
2008	231.1	163.7	11.3	9.6	5.7	9.3	5.5	4.2	356.7	315.1	27.7	23.8	10.7	9.5
2009	255.5	234.0	11.7	13.0	6.9	7.2	2.9	1.9	380.3	550.6	28.7	34.5	9.4	15.2
2010	205.5	160.8	12.0	12.4	7.9	7.2	3.8	2.5	420.1	454.8	27.6	29.8	12.4	13.3
2011	193.1	119.0	12.6	14.2	7.0	6.7	3.6	1.0	386.9	662.0	26.8	44.6	10.8	19.9
2012	215.5	105.4	12.4	14.4	7.7	6.9	3.0	1.1	307.8	592.7	26.6	35.1	7.0	16.7
2013	234.2	232.8	10.4	12.3	11.6	8.4	6.7	3.9	294.8	566.4	22.9	35.1	8.6	18.9
2014	191.5	216.8	13.4	10.6	6.9	7.5	2.4	2.2	220.9	329.5	27.9	29.8	6.6	11.8
2015	201.2	174.5	12.6	11.8	7.3	7.8	3.8	2.7	362.2	460.1	27.1	30.4	9.5	13.2
2016	201.0	201.0	13.0	13.0	6.0	6.0	3.1	3.1	496.4	496.4	42.4	42.4	13.0	13.0
2017	176.3	131.4	11.1	13.2	9.6	7.9	4.8	1.6	305.5	517.2	25.4	38.1	8.5	17.1
2018	285.0	251.4	10.5	12.3	9.5	7.2	5.5	2.9	285.8	463.9	32.1	37.1	10.3	14.5
2019	195.8	119.9	11.3	14.0	9.2	7.3	4.6	1.6	314.2	401.4	24.8	25.3	9.4	12.9
2020	227.7	208.0	11.3	12.9	6.8	7.4	3.4	2.5	215.0	426.3	15.0	29.7	5.1	12.9
Mean Std	216.4 29.8	178.4 49	11.8 0.9	12.6 1.4	7.9 1.7	7.4 0.8	4.1 1.3	2.4 1.0	334.4 79.9	479.7 100.0	27.3 6.0	33.5 6.2	9.3 2.2	14.5 2.9

The analysis of the relationships between grape composition and climate variables obtained with the factor analysis after the varimax rotation is shown in Table 2. Four factors were retained, which explained 85% of the variance. It can be observed that the highest weight in the first factor, which explained 37.35% of the variances, was for the variables related to the phenolic components, with positive sing, and for the berry weight and malic acid, with negative signs. However, the coefficients for the variables related to temperature and precipitation were very small. The highest values in the second factor, which explained 18.18% of the variance, corresponded to berry weight, titratable acidity and malic acid and the variables that represented the ASW in the periods SF-V and V-H, being all of them positive. The third factor, which explained 16.27% of the variance, was mainly driven by the temperatures recorded in the ripening period, and the highest values in the fourth factor, which explained 13.75% of the variance, were related to titratable acidity (negative values) and temperatures referred to the period SF-V, which had positive sign.

**Table 2:** Results of factor analysis obtained for Carignan combining grape composition and variable referring maximum and minimum temperatures and soil water available in two periods between separated flowers and maturity (included values from 2008 to 2020 of two plots in Rioja DOCa) (*BW-100b*, weight of 100 berries; *AcT*, titratable acidity; *AcM*, malic acid; *AntT*, concentration of anthocyanins; *TPI*, total polyphenol index; *CI*, colour intensity; *Tmax*, maximum temperature; *Tmin*, minimum temperature; *ASW*, available soil water; *FS-V*, flowers separated-veraison period; *V-H*, veraison to harvest period).

	Factor 1	Factor 2	Factor 3	Factor 4	Commun	*Factor*	*Eigenvalue*	*Percent. variance*
BW-100b	** − 0.559**	**0.609**	− 0.141	0.126	0.72	1	4.48	37.35
AcT	− 0.034	**0.575**	0.181	** − 0.450**	0.57	2	2.18	18.18
AcM	− **0.635**	**0.610**	− 0.211	− 0.176	0.85	3	1.95	16.27
AntT	**0.920**	− 0.240	− 0.005	0.150	0.93	4	1.64	13.65
TPI	**0.834**	0.172	**0.357**	− 0.014	0.85	5	0.79	6.58
CI	**0.973**	0.032	0.095	− 0.012	0.96	6	0.45	3.77
Tmax FS-V	0.050	− 0.248	0.117	**0.914**	0.91	7	0.20	1.69
Tmin FS-V	0.033	0.231	0.054	**0.914**	0.89	8	0.11	0.93
Tmax V-H	0.156	− 0.066	**0.948**	− 0.044	0.93	9	0.10	0.81
Tmin V-H	0.177	− 0.066	**0.954**	0.150	0.97	10	0.06	0.49
ASW FS-V	− 0.096	**0.706**	− 0.437	− 0.278	0.78	11	0.03	0.25
ASW V-H	0.093	**0.921**	0.022	0.214	0.90	12	0.01	0.05
Bold numbers show the variables that have higher loadings in each factor								

The additional stepwise forward regression analysis done considering these variables showed that both acidity and anthocyanins were related to the ASW (Table SM1). This result agrees with that observed for acidity in the PCA (factor 2), but the effect on anthocyanins seemed to linked to the dilution effect that produces the higher berry weight associated to the higher water availability. However, the effect of temperature on titratable acidity found in factor 4 (which was the one that explained lower percentage of the variance) seemed to be hidden by the effect of the water effect. The ASW in the period V-H gave significant fits for AcT, while malic acid and anthocyanins were related to ASW in the period FFS-V. For AcT and AcM, the correlation coefficient was positive, while for AntT, the coefficient was negative. Nevertheless, the explained variance was quite small 13, 29 and 11%, for AcT, AcM and AntT, respectively).

### Projected changes in climatic variables and their impacts

#### Projected changes in temperature and precipitation

The changes in monthly maximum and minimum temperature and precipitation, projected using the ensemble of models for 2050 and 2070 under the scenarios analysed, are shown in Table SM2. During the months corresponding to the growing season (April–October), the increase in the average Tmax under the scenario RCP4.5 could be of about 1.4 °C for 2050 and 1.8 °C for 2070, and under the RCP8.5 scenario could be near 2 and higher than 3 °C, for 2050 and 2070, respectively. The projected increase in Tmin during the growing season by 2050 could range between near 1 °C under the RCP4.5 scenario and 1.5 °C under the RCP8.5 scenario, and it could be of up to 2.5 °C by 2070. A decrease in precipitation is projected under both scenarios, which regarding the growing season could range between 10 and 16% by 2050, respectively under the RCP4.5 and RCP8.5 scenarios, and up to 30% by 2070, under the warmest scenario. This means that the growing season precipitation, which is already scarce at present, will be quite small, leading to greater water deficits.

#### Projected changes in vine phenology for Carignan in the study area

Based on the thermal requirements observed during the period 2008–2020 to reach each phenological stage (GDD values) and the projected changes in temperature, an advance in the phenological timing was projected (Table 3). Under both scenarios, the projected advance appears to be greater for maturity and veraison than for flowers separated. For flowers separated, the advance by 2050 could vary between 4 and 7 days respectively under the RCP4.5 and RCP8.5 scenarios, while veraison is projected to be advanced 6 and 12 days, and maturity could be advanced between 11 and 17 days, respectively, under the same scenarios. By 2070 and under the warmer scenario, the advance could be near double.

**Table 3:** Projected changes in phenology and in grape composition for Carignan in Rioja DOCa by 2050 and under warmer scenarios (RCP4.5 and RCP8.5). *FS*, flowers separated; *V*, veraison; *BW-100b*, weight of 100 berries; *AcT*, titratable acidity; *AcM*, malic acid; *AntT*, concentration of anthocyanins)

Scenario	Phenology			Grape composition			
	Stage H (FS) (days)	Stage M (V) (days)	Maturity (days)	BW-100b (g)	AcT (g/L)	AcM (g/L)	AntT (mg/L)
RCP4.5 2050	− 4 ± 1.4	− 6 ± 2.4	− 11 ± 1.0	− 26	− 0.26	− 0.09	3.7
Change (%)				− 12.9	− 3.4	− 3.0	0.9
PCP4.5 2070	− 6 ± 0.7	− 11 ± 2.5	− 14 ± 0.6	− 37	− 0.37	− 0.14	6.1
Change (%)				− 18.4	− 4.8	− 4.9	1.5
RCP8.5 2050	− 7 ± 1.4	− 12 ± 1.8	− 17 ± 1.0	− 33	− 0.34	− 0.14	5.8
Change (%)				− 16.7	− 4.4	− 4.7	1.4
PCP8.5 2070	− 13 ± 0.7	− 19 ± 2.5	− 26 ± 2.7	− 58	− 0.58	− 0.28	11.88
Change (%)				− 28.5	− 7.5	− 7.5	2.9

#### Projected changes in grape composition

Taking into account the relationship between grape composition and the climatic variables that presented significant fits and the projected changes in temperature and precipitation under different emission scenarios, change projections in grape composition were made. The projected changes in berry weight, acidity and phenolic compounds are shown in Table 3. Berry weight could suffer reductions of between 26 and 33 g/100 berries by 2050 under the RCP4.5 and RCP8.5 scenarios, respectively, which represent a reduction of about 12.9 to 16.7%, depending on the scenario. That projected change, nevertheless, is smaller than the variability observed during the period analysed. Based on the reduction on precipitation along the growing cycle and the observed relationship between acidity and water availability, titratable acidity may suffer reductions between 0.26 and 0.34 g/L while malic acid concentration could undergo r very small reductions (between 0.1 and 0.14 g/L by 2050, and between 0.14 and 0.28 g/L by 2070 respectively under the RCP4.5 and RCP8.5 scenarios). The concentration of anthocyanins could, however, increase slightly due to increasing water deficits (in about 3.7 and 5.7 mg/L by 2050 under the RCP4.5 and RCP8.4 emission scenario, respectively.

## Discussion

Despite the variability in the weather conditions recorded from year to year in the period analysed, significant trends in Tmax and Tmin referring the vine growing season were observed (0.13 °C and 0.15 °C per year for Tmax and Tmin, respectively). These trends are of the same order of magnitude to those recorded for the same period in other areas (Ramos et al. 2021), but greater than the average trend for longer series that include the last few decades (Ramos et al. 2015). The results are in agreement with the warmer conditions in many years of the last decade and already pointed out around the world (NOAA National Centers for Environmental Information 2021). Regarding precipitation, the high variability from year to year, hidden any trend. The remarkable result was the scarce precipitation recorded in the area during the growing season, which represented less than 40% of annual precipitation. The precipitation recorded during the ripening period was very scarce, which produce significant water deficits at the end of the cycle. The ASW reached very low values during the ripening period in most of analysed years (< 5% of SWC) although there were differences between years and also between plots (Figure SM2). The smallest differences between plots were recorded in years like 2011, 2012 or 2017, which were years in which high temperatures were recorded. In those years, low ASW values (< 20% of SWC) were also found in earlier stages within the growing cycle, which meant that the vines suffered severe stress.

The phenological timing showed high variability between years, with differences of up to 19 days for flowers separated, up to near 1 month for veraison and up to 40 days for harvest. Despite the variability from year to year, there was a trend of advance in veraison and harvest of about 0.9 days/year, which means about 10 days on average in the period analysed. However, there were no significant trends for the stage flowers separated. As it was previously commented, this variety has a late budding and late ripening. It was confirmed that for the same area of Rioja, and in zones located at the same elevation, flowers separated dates for the Carignan did not differ so much of the phenological dates of the main variety cultivated in the area (Tempranillo) and there were also similar to those for Grenache. However, there were significant differences for veraison and maturity between Carignan and Tempranillo, being in the study case not only later than Tempranillo but also later than Grenache. Information for these two varieties in plots close to P2 can be seen in Ramos and Martínez de Toda 2020b, and for Tempranillo in plots close to P1 is shown in Ramos et al. 2020. Although an advancing trend of veraison and maturity was observed in the last years, there were some specific years with particularly advanced phenology (2017 or 2011), which corresponded to years with high temperatures in relation to the average. This fact may be considered an example of what could be the vine response under warmer scenarios. The results of the relationships between phenological timing and climate variables indicated that the dominant factor leading to changes in the beginning and end points of the plant phenophase was temperature (Table SM1). Despite the fact that later phenological dates were observed in the wettest years, which were also the ones with lower temperatures, there was not a significant effect of the ASW on the phenological timing. Only the ASW in the period between flowers separated and veraison showed a significant effect at 90% on maturity. Thus, temperature seems to be the main driver of the variability in the phenological timing.

The projected advances in phenology for Carignan (Table 3) were of the same order of magnitude of projected changes in other areas and for other red grape varieties (Cabernet Sauvignon, Tempranillo, Grenache, Merlot, Pinot Noir) (Pieri et al. 2012; Cuccia et al. 2014; Fraga et al. 2016; Hall et al. 2016; Ramos and Jones 2018; Alikadic et al. 2019). For the same study area (Rioja DOCa and the same elevation), the projected advances for Carignan in relation to those for Tempranillo presented small variations: 5, 8 and 11 days vs. 5, 9 and 14 days, by 2050 under the scenario RCP4.5, and 7, 12 and 17 vs. 7, 11 and 19 days, by 2050 under the RCP8.5 scenario for flowers separated, veraison and maturity, respectively (Ramos and Martínez de Toda 2020a). However, it is necessary to remark that although the differences could be small, those phenological stages, and in particular veraison and harvest, are already earlier for Tempranillo than for Carignan, which means ripening under warmer conditions for Tempranillo than for Carignan. This means that the effect on grape composition could be different.

The earlier ripening implies that the process occurs under warmer conditions and it could also affect the grape composition. This risk has been indicated for different varieties and authors. In this respect, van Leeuwen et al. (2019) pointed out the risk of reaching too elevated sugar contents due to the acceleration of the processes in the grape by the increasing temperatures. However, the variety considered in this research is a late ripening variety that, under the present conditions in Rioja DOCa, does not reach its maximum potentiality. The probable alcoholic degree in many years did not reach the 13° for Carignan (during the period of study, the average PVAD was 11.8° in plot P1 and 12.6° in plot P2) while for other varieties like Tempranillo, that value is reached and over passed in all zones of the Rioja DOCa. Thus, higher temperature accumulation could favour an increase in sugar content.

Regarding other parameters like acidity and anthocyanins, the small effect of temperature on grape composition was confirmed in the multivariate analysis carried out, which pointed out, however, the effect of water availability in the period FS-V and V-H on grape composition as dominant factor. The results indicated a decrease in titratable acidity with increasing maximum temperatures, which would agree with results found for other red varieties cultivated in the same area (Ramos and Martínez de Toda 2020b) and with the idea that under warmer temperatures grapes will have lower acidity. However, no effect was confirmed on malic acid, which is considered the acid that could suffer more under warmer temperatures (Picariello et al. 2019; Arrizabalaga-Arriazu et al. 2020). In addition, the ratio AcM/AcT did not show any trend during the period analysed, with values similar to the average at harvest in the hottest years (2011, 2012 or 2017), although these ratios were always greater in plot P1 than in plot P2. However, that ratio was higher in the wettest years (2008, 2013 and 2018). The results obtained in this study showed that for this variety, when either temperature and water availability were considered together, the effect of temperature on acidity was not significant or it was hidden, being the effect of the water availability the dominant effect. The titratable acidity presented a negative relationship with maximum and minimum temperatures recorded before veraison. However, the effect of temperature on malic acid was negligible. This will mean, that under the projected climate change scenarios, with a decreasing trend in precipitation, a slight decrease in acidity could occur, but it could be relatively small (Table 3). That decreasing trend would represent a reduction in relation to the present lesser than 5% by 2050 under the RCP4.5 scenario, and up to 7.5% by 2070 under the warmer scenario.

Regarding the concentration of anthocyanins, which could be another parameter susceptible to decrease under increasing temperature (Bergqvist et al. 2001; Teixeira et al. 2013), the results obtained in this research did not show any significant effect of temperatures on phenolic compounds. This result represents a different behaviour of Carignan in relation to other varieties cultivated in the area (Ramos and Martínez de Toda 2020b). As it was previously mentioned, the concentration of anthocyanins of this variety is smaller than for other cultivars in the area. For example, for the same period analysed, the AntT for Tempranillo were 452 ± 72 and 532 ± 98 mg/L respectively for the zones where P1 and P2 were located, while for Carignan, the average AntT were 334 ± 78 and 480 ± 100 mg/L, respectively, for the same zones. The concentration of anthocyanins increased with the accumulated temperatures during the ripening period, and even under the warmer conditions suffered in the years 2011, 2012 or 2017, the increase continued. This fact contrasts with the effect of heat accumulation on other varieties like for Tempranillo observed under warm conditions (Chacón-Vozmediano et al. 2021), which indicate that they had not reach the maximum value. The ratio Ant/PVAD in those years was not smaller than in cooler years. Even more, that ratio, in the plot P2, which always had higher values than plot P1, showed higher values at harvest than the average in those hottest years (2011, 2012 or 2017). This means that, for Carignan, being a late ripening variety, the increase in temperature could still favour the increase in sugar content and in anthocyanins, without any decoupling. The Ant/PVAD is still lower than for other red varieties in the same viticultural area (Ramos and Martínez de Toda 2020a).

The only effect observed in this analysis, which was similar for the anthocyanins, the TPI and CI, was their relationship with water availability indicating an increase when water stress increased, which in addition produces a decrease in berry size (Table 3). In addition, the first factor obtained in the factor analysis confirmed lower concentration of anthocyanins and phenolic compounds with increasing berry weight by dilution effect. Thus, the changes in the phenolic compounds that could be expected would be mainly associated to water availability. The effects of water deficits on anthocyanins and phenolic compounds have been previously confirmed in other red varieties such as Grenache, Cabernet Sauvignon or Tempranillo (Castellarin et al. 2007; Fernandes de Oliveira et al. 2013; Chacón-Vozmediano et al. 2021) and the results also agree with the decrease in berry mass associated to water stress at ripening observed by Fernandes de Oliveira and Nieddu (2016) in Carignan. In the study case, berry weight was mainly influenced by water availability during the ripening period (which reached very low values—below 5% of SWC in most of the analysed years), but also in the period FS-V, which were also quite low.

Under the projected decrease in precipitation, higher water stress could appear in those periods, which could contribute to a slight decrease in berry weight and to an increase in the anthocyanins in agreement with the observed results (Table 2). According to the projected changes in precipitation, the decrease in berry size by 2050 could range between 12.9 and 16.7% in relation to the present, depending on the scenario and the change in anthocyanins could be up to 12 mg/L, taking into account only water reduction. This would mean a change lesser than 5%, but it could increase due to the decrease of the dilution effect as berry size will also decrease and, in addition as it was observed, the increase in temperature accumulation can also contribute to increase both sugar and anthocyanins.

## Conclusions

The response of the Carignan variety cultivated in the central part of the Rioja DOCa, under a wide range of weather conditions, allowed extracting information about what are the main climatic drivers for this variety, and how it could be affected under warmer scenarios. The phenological timing, in particular veraison and maturity, is mainly driven by temperature recorded during the growing season. By 2050, veraison is projected to experience an advance of about 6 and 12 days, while for harvesting the projected advance range between 11 and 17 days under the RCP4.5 and RCP8.5 scenarios, respectively. These advances, which are smaller than the ones projected for other majority varieties in the area, show the potentiality of Carignan to be grown in the area.

Being Carignan a late ripening variety and producing at present wine of higher acidity, which has made this variety until now a good complement to Tempranillo, it is a variety that could be affected, regarding acidity, in less proportion than Tempranillo under warming scenarios and with less available water. Available water will also affect other grape parameters such as berry weight and anthocyanins. The impact will be an increase in the concentration of anthocyanins which, in addition, will be favoured by the warmer conditions, under which both sugar content and anthocyanin could still increase without a decoupling effect. The results obtained in this research for Carignan, which differ from those obtained for other red varieties, as it is the case of Tempranillo, although they are limited to one area within the Rioja DOCa, contribute to the knowledge on the strategies to be adopted in the face of warming scenarios.

## Supplementary Information

Below is the link to the electronic supplementary material.

Supplementary file1 (DOCX 446 KB)

## Data Availability

Not applicable.
