# Supplementary material for: Influence of weather conditions and projected climate change scenarios on the suitability of Vitis vinifera cv. Carignan in Rioja DOCa, Spain
Source: Int J Biometeorol. 2022 Mar 11;66(6):1067–78. doi: 10.1007/s00484-022-02258-6 (PMC9132826; doi:10.1007/s00484-022-02258-6)
Supplement: Supplementary file 1 — Supplementary file1 (DOCX 446 KB) [file 484_2022_2258_MOESM1_ESM.docx]

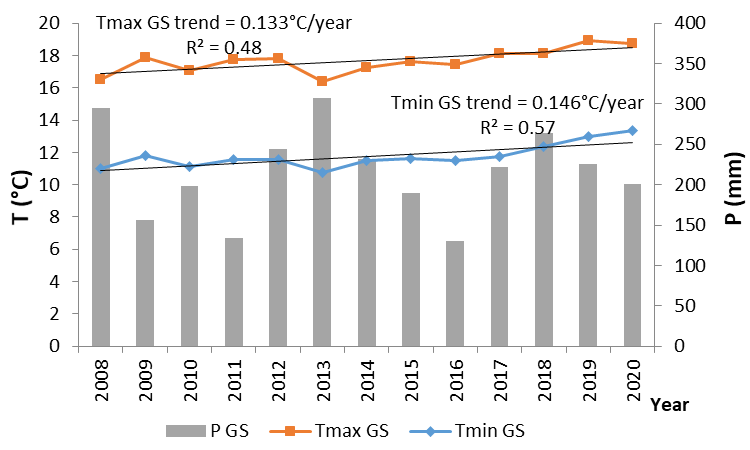


Figure SM1. Growing season (April 15^th^- October 15^th^, on average) maximum and minimum temperature and precipitation recorded during the period of study (2008-2020) in Logroño LG weather station.


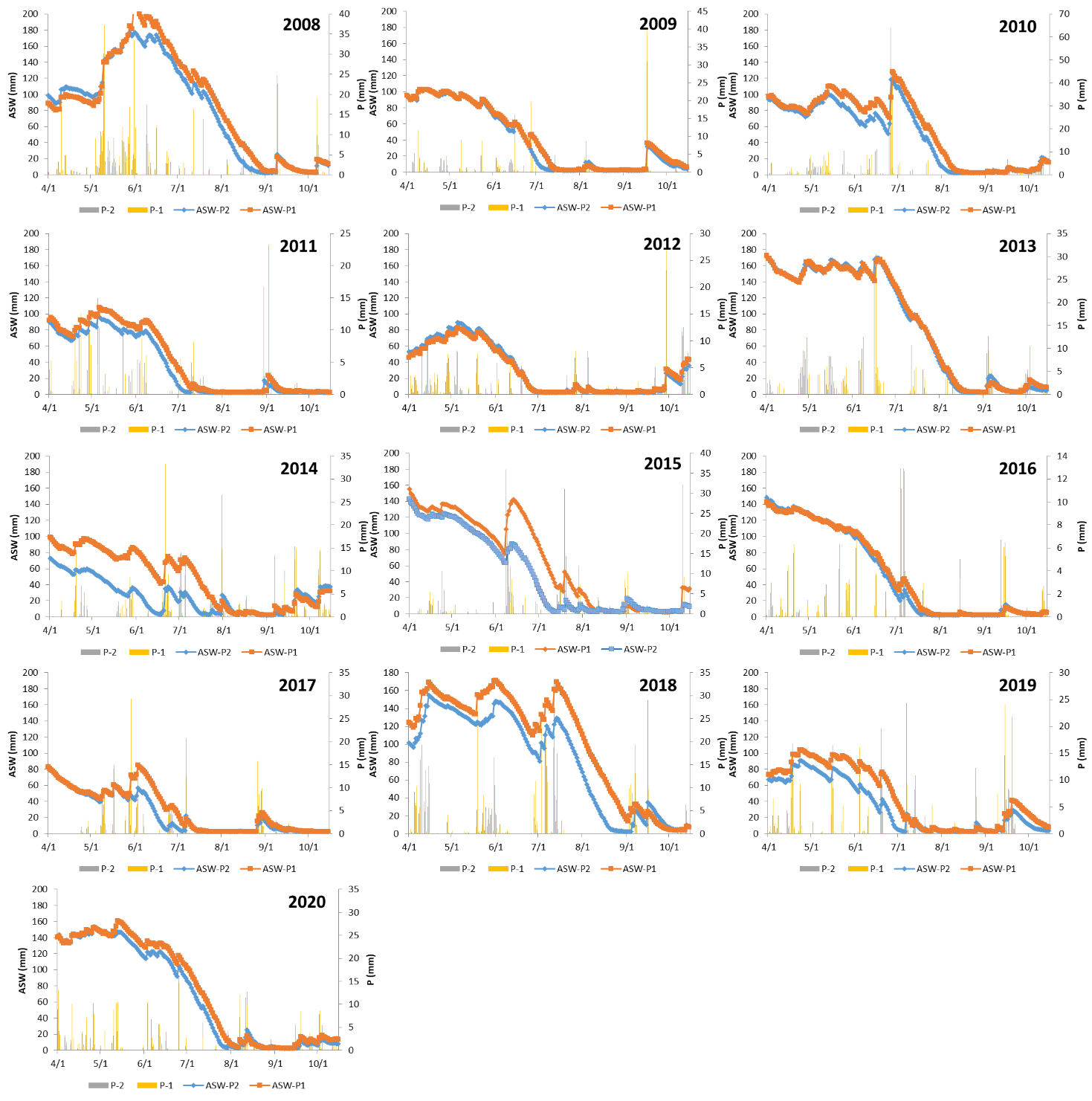
 Figure SM2. Available soil water in both plots estimated for each of the years analysed during the growing season period.

Table SM1: Significant relationships in the stepwise forward multiple regression analysis (and correlation coefficients) between phenological dates and grape composition with climate parameters referring to different periods, based on the values recorded in the period 2008-2020 in two plots of the Rioja DOCa. (BB-FS: period budbreak to flowers separated; FS-V: period flowers separated-veraison: V-H: period veraison to harvest; BW-100b: weight of 100 berries; AcT: titratable acidity; AcM. Malic acid; AntT; concentration of anthocyanins).

|  | Tmax  BB-FS | Tmin  BB-FS | Tm  BB-FS | | | Tmax  FS-V | | Tmin  FS-V | | Tm  FS-V | | Tmax  V-H | Tmin  V-H | | Tm  V-H | | ASW  BB-FS | | ASW  FS-V | | ASW  V-H |
| --- | --- | --- | --- | --- | --- | --- | --- | --- | --- | --- | --- | --- | --- | --- | --- | --- | --- | --- | --- | --- | --- |
| Stage H (FS) | ns | ns | ns | | |  | |  | |  | |  |  | |  | | ns | |  | |  |
| Stage M (V) | ns | -2.77*  (0.29) | -3.87***  (0.63) | | | -2.1*  (0.21) | | -3.28*  (0.21) | | -2.73*  (0.25) | |  |  | |  | | 0.11*  (0.28) | | 0.09*  (0.25) | |  |
| Harvest | ns | -3.39*  (0.23) | -4.99***  (0.54) | | | -3.2*  (0.25) | | ns | | -3.27*  (0.18) | | -3.26*  (0.29) | -5.0*  (0.32) | | -3.65*  (0.32) | | 0.13*  (0.33) | | 0.18*  (0.41) | | ns |
| BW-100b |  |  | |  |  | |  | |  | |  | |  |  | |  | |  | | 7.19***  (0.32) | |
| AcT |  |  | |  | ns | | ns | |  | | ns | | ns |  | |  | |  | | 0.015*** (0.14) | |
| AcM |  |  | |  | ns | | ns | |  | | ns | | ns |  | |  | | 0.023*** (0.32) | |  | |
| AntT |  |  | |  | ns | | ns | |  | | ns | | ns |  | |  | | -0.089*** (0.11) | |  | |

ns: no significant; *: significant al 90%; **: significant at 95%; ***: significant at 99%.

Table SM2. Average monthly projected changes in maximum and minimum temperature and precipitation for 2050 and 2070 under the RCP 4.5 and RCP 8.5 scenarios (obtained using an ensemble of models) for the study area.

|  |  | **Jan.** | **Feb.** | **Mar.** | **Apr.** | **May** | **June** | **July** | **Aug.** | **Sept.** | **Oct.** | **Nov.** | **Dec.** |
| --- | --- | --- | --- | --- | --- | --- | --- | --- | --- | --- | --- | --- | --- |
| **RCP4.5 2050** | ΔTmax | 0.9 | 0.7 | 1.1 | 1.1 | 1.4 | 1.8 | 1.8 | 1.6 | 1.7 | 1.4 | 0.9 | 0.9 |
|  | ΔTmin | 0.8 | 0.7 | 0.8 | 0.7 | 0.8 | 1.2 | 1.4 | 1.3 | 1.3 | 1.1 | 0.8 | 0.8 |
|  | ΔP | -3.2 | 2.1 | -3.7 | -3.2 | -3.5 | -5.2 | -3.0 | -4.9 | -2.9 | -4.4 | 0.6 | -1.3 |
| **PCP4.5 2070** | ΔTmax | 1.1 | 1 | 1.4 | 1.4 | 1.8 | 2.3 | 2.4 | 2.2 | 2.2 | 1.7 | 1.3 | 1.2 |
|  | ΔTmin | 1 | 0.8 | 1 | 0.9 | 1.1 | 1.6 | 1.8 | 1.7 | 1.8 | 1.4 | 1 | 1 |
|  | ΔP | -1.9 | 0.5 | -3.4 | -3.4 | -7.7 | -8.4 | -5.4 | -5.8 | -4.8 | -4.9 | 1.5 | -0.5 |
| **RCP8.5 2050** | ΔTmax | 1.3 | 1.2 | 1.5 | 1.7 | 1.9 | 2.5 | 2.5 | 2.4 | 2.5 | 2 | 1.5 | 1.4 |
|  | ΔTmin | 1.2 | 1 | 1.1 | 1.1 | 1.3 | 1.9 | 2.1 | 2 | 2.1 | 1.6 | 1.3 | 1.2 |
|  | ΔP | -2.2 | 1.3 | -5.1 | -6.4 | -7.1 | -6.6 | -6.4 | -5.9 | -4.0 | -6.9 | -1.0 | 2.0 |
| **RCP8.5 2070** | ΔTmax | 2.1 | 2.1 | 2.3 | 2.7 | 3.2 | 4.1 | 4.4 | 4.1 | 4.1 | 3.2 | 2.5 | 2.3 |
|  | ΔTmin | 1.8 | 1.8 | 1.8 | 1.9 | 2.1 | 3 | 3.5 | 3.3 | 3.4 | 2.7 | 2.1 | 1.9 |
|  | ΔP | -5.3 | 0.5 | -7.0 | -10.7 | -13.9 | -14.8 | -12.7 | -10.7 | -6.5 | -10.4 | -3.4 | -1.5 |
